# How Peru introduced a plan for comprehensive HIV prevention and care for transwomen

**DOI:** 10.7448/IAS.19.3.20790

**Published:** 2016-07-17

**Authors:** Ximena Salazar, Arón Núnez-Curto, Jana Villayzán, Regina Castillo, Carlos Benites, Patricia Caballero, Carlos F Cáceres

**Affiliations:** 1 Center for Interdisciplinary Studies in Sexuality, AIDS and Society, Universidad Peruana Cayetano Heredia, Lima, Peru; 2 RedTrans-Perú, Lima, Peru; 3 UNAIDS, Office for the Andean Region, Lima, Peru; 4 Ministerio de Salud del Perú, Lima, Peru; 5 Instituto Nacional de Salud del Perú, Lima, Peru

**Keywords:** HIV prevention, transwomen, evidence synthesis, structural vulnerability, policy dialogue, Peru

## Abstract

**Introduction:**

As a group, transwomen in Peru have the highest prevalence of HIV (>20%) in the country, but they have little access to HIV prevention, testing and care services. Until recently, Peru's national HIV programme did not recognize transwomen and had remained essentially static for decades. This changed in December 2014, when the Ministry of Health expressed its commitment to improve programming for transwomen and to involve transwomen organizations by prioritizing the development of a “Targeted Strategy Plan of STIs/HIV/AIDS Prevention and Comprehensive Care for Transwomen.”

**Discussion:**

A policy dialogue between key stakeholders – Peru's Ministry of Health, academic scientists, civil society, transgender leaders and international agencies – created the conditions for a change in Peru's national HIV policy for transwomen. Supported by the effective engagement of all sectors, the Ministry of Health launched a plan to provide comprehensive HIV prevention and care for transwomen. The five-year plan includes new national guidelines for HIV prevention, care and support, and country-level investments in infrastructure and equipment. In addition to new biomedical strategies, the plan also incorporates several strategies to address structural factors that contribute to the vulnerability of transwomen. We identified three key factors that created the right conditions for this change in Peru's HIV policy. These factors include (1) the availability of solid evidence, based on scientific research; (2) ongoing efforts within the transwomen community to become better advocates of their own rights; and (3) a dialogue involving honest discussions between stakeholders about possibilities of changing the nation's HIV policy.

**Conclusions:**

The creation of Peru's national plan for HIV prevention and care for transwomen shows that long-term processes, focused on human rights for transwomen in Peru, can lead to organizational and public-policy change.

## Introduction

### Research and the recognition of transwomen

In many parts of the world, transwomen are nearly 50 times more likely to acquire HIV than are members of the general population [1]. In Peru, the HIV epidemic is concentrated among men who have sex with men (MSM) and transwomen, who were originally grouped within the MSM category. Targeted studies [2,3] have shown that transwomen in Peru have a high prevalence of HIV (24–30%); their vulnerability is especially evident in the capital city of Lima, where transwomen have an HIV prevalence of 30%, compared to 0.23% among the general population [2] and 12.4% among the MSM in the country [3].

In general, the high incidence of HIV among transwomen has been linked to biological risks (e.g. receptive anal sex), behavioural risks (e.g. condom-less sex, a high number of partners and transactional sex) and structural factors (e.g. discrimination and no legal recognition of their gender identity) [4–6]. These factors are also evident among the population of transwomen in Peru, estimated to be nearly 23,000 in 2010 [7]. Violence against transwomen, often perpetrated by government security forces, is not uncommon in Peru [8,9]. In Lima, the majority of transwomen come from poor or extremely poor households, nearly half (46%) are between 25 and 35 years old, and nearly one in five (18%) migrated to Lima from the Peruvian Amazon to engage in sex work [2]. The absence of laws, policies and regulations that recognize transwomen exacerbates their social and structural exclusion [2,10–12].

All of these factors contribute to a high unmet need for HIV prevention, care and support for transwomen in Peru [13–16]. Indeed, until recently, Peru's national HIV programme did not recognize transwomen and had remained essentially static for decades. In the mid-1990s, two important strategies increased the visibility of vulnerable populations: (1) a programme of periodic medical check-ups for sex workers and MSM and (2) a strategy that trained peer-group members to promote safer sex. Another important step was taken in 2004 with the launch of a national antiretroviral treatment programme that ensured free access to antiretroviral therapy for the entire population. In the ensuing years, transwomen were still considered to be MSM, but the community had begun to mobilize and demand the recognition of their gender identity.

### The role of national consultations

Although transwomen organizations have existed since the early 2000s, it was not until 2007 that a distinct transwomen social movement started to gain some visibility. This was manifested by the first “National Consultation on Sex Work, Human Rights and HIV and AIDS” before the year ended. The discussions during the Consultation considered the social and health situations of transwomen, and the structural determinants of their vulnerability to HIV/AIDS [13,17].

The 2007 Consultation also helped to initiate a collaboration (which lasted until 2012) between transwomen community organizations, UNAIDS, UNFPA and academic researchers [13]. These collaborations had two major components: (1) developing the transwomen community and building the capacity of transwomen organizations throughout Peru and (2) increasing the awareness of decision-makers and authorities in several Peruvian cities about the plight of transwomen. As a result of these collaborations, the national transwomen movement became a stakeholder in HIV and human rights issues, and authorities became aware of the movement's claims [13].

By 2014, it became evident that Peru's response to HIV among MSM and transwomen needed an update that included a combination of prevention strategies [17]. The Ministry of Health organized a “National Consultation on Combination HIV Prevention,” in partnership with Cayetano Heredia University (UPCH) and UNAIDS. This 2014 Consultation was an important milestone that opened a dialogue between stakeholders and community leaders on the future of HIV prevention in Peru. The dialogue resulted in a broad set of recommendations, including (1) HIV-prevention programmes should meet the particular needs of each key population, including the different modes of HIV transmission among these groups; (2) The assessment of a study's outcome must consider structural interventions (e.g. law reform and enforcement to eliminate stigma, discrimination and violence); (3) Health providers need a better understanding of how transwomen perceive health and the barriers they face in accessing health services; and (4) The support of grassroots organizations and the transwomen community should be a critical part of the design and implementation of health care strategies.

### A new plan

In December 2014, the Ministry of Health expressed its commitment to improve programming for transwomen and involve transwomen organizations by prioritizing the development of a “Targeted Strategy Plan of STIs/HIV/AIDS Prevention and Comprehensive Care for Transwomen.” The national HIV/STI programme at the Ministry of Health formed a working group, experts from the Ministry, academia, UNAIDS and transwomen organizations, who were important contributors to the new plan.

The plan established the following goals: (1) strengthen the diagnostic and treatment capacity of the referral centres to provide primary care to transwomen; (2) establish a specific technical and programmatic policy framework to address the primary health needs of transwomen; (3) create two centres of excellence to provide comprehensive care for transwomen; (4) strengthen information management and health monitoring and surveillance of transwomen; (5) provide access to STI/HIV diagnostic, treatment and care services for transwomen; and (6) strengthen the participation of transwomen organizations.

The working group's first action was to map the social space of transwomen in the cities of Lima and Callao with the aim of describing the population's geographical distribution, identifying problems and understanding the relationships between transwomen and members of other populations. The results of the mapping played an important role in the assessment of Peru's HIV strategy and helped to define a plan to reduce the transmission of HIV/STIs among transwomen in Lima and Callao.

Some other activities are also planned for the near future. The capacity of health services will be improved by regulations that address the primary health needs of transwomen according to current scientific evidence and the recommendations by the World Health Organization. Transwomen will gain greater access to health services through the implementation of mobile health care services and improvements to health facility infrastructures. Two centres of excellence will implement a comprehensive approach to health care for transwomen by providing STI/HIV/AIDS prevention and control, mental health services, cancer prevention and medically supervised hormone therapy for body modifications. These centres will collaborate with public and private specialized institutions, and with the participation of transwomen organizations and community groups.

## Discussion

The development of Peru's HIV plan for transwomen shows that significant changes in public policy can be achieved by the concerted (and long-term) efforts of researchers, transwomen and policy makers. We identified three key elements in this process: (1) the availability of relevant data from a variety of sources, including strategic data to support programmatic change; (2) ongoing strengthening of transwomen communities through capacity building for advocacy and political participation; and (3) a policy dialogue to identify and address the needs of transwomen in a feasible and sustainable way.

Public health care initiatives for transgender people have been implemented in some other Latin American countries. For instance, specialized centres for transgender health and “friendly” health care centres in public hospitals have been implemented in Mexico, Argentina and Uruguay, with the aim of providing comprehensive health care and serving as bridges to other health systems services [18,19]. Brazil and Argentina have developed specific policies that guarantee access to health care for LGBT people, and so provide a normative framework based on evidence and human rights [20,21].

As specialized health care centres for transwomen are created, the health care system could be adapted to address the needs and demands of transwomen by developing guidelines and training health providers [22]. Policy development and community involvement are key elements of change because normative frameworks are needed to move from well-intentioned initiatives to public commitments, while the community's participation helps to create health services that are grounded in the community's needs and expectations.

It is clear that HIV-prevention programmes for transwomen must tackle the stigma that affects their lives and increases their vulnerability to HIV. A structural approach to stigma analyses the process of stigmatization at the junction of culture, power and difference [23], and it recognizes that social conditions and institutional policies and practices limit the opportunities and resources among stigmatized people [24,25]. For transwomen, the stigma associated with HIV is intertwined with the stigma of a non-conforming gender identity. This is evident in the relationship that the Peruvian government has established with the transwomen community, which is firmly defined by HIV epidemic.

Political leaders and the transwomen community do agree that the health strategies for transwomen must go beyond HIV prevention and care, and address transwomen's demands for comprehensive care. An HIV-prevention strategy tailored to transwomen must address the dual forms of stigma they encounter, while finding a balance between addressing HIV and the population's other needs. It must also promote fundamental changes in the legal and social contexts that make transwomen so vulnerable to HIV [26].

It may be useful to consider the experience of “friendly” health centres in Argentina and the health centres that are free from “homo-lesbo-trans-phobia” in Uruguay [18,19], both of which offer comprehensive health care. These centres were originally part of HIV and STI programmes, but they distanced themselves from the “HIV label” because the communities do not want to be identified solely as a key population. The recognition of this need is critical and denying its importance may be a barrier that prevents transwomen from trusting the health system. It may be feasible to use HIV/STI services as an entryway to integrate transwomen within the health system, but it is only a first step in a complex process toward comprehensive health care for the community.

The sustainability of any programme for transwomen in Peru is also an important consideration. The realm of politics in Peru is highly unstable, and several stakeholders have identified this issue as the primary barrier to the implementation of a combination prevention programme for transwomen and MSM. Discriminatory attitudes, fears and poor support have typified the government's past responses to the demands of transwomen and MSM [27,28]. An effective HIV policy must be reliable, and it must be greeted with a consensual agenda from the transwomen community. The government's new HIV policy for transwomen is an important step, but its sustainability depends on how well it can be integrated into the nation's broader policy on HIV and how it will evolve during future administrations.

## Conclusions

Evidence-informed political will and multisector policy dialogue were key elements in the creation of the “Targeted Strategy Plan of STIs/HIV/AIDS Prevention and Comprehensive Care for Transwomen” in Peru. The active participation of diverse stakeholders in policy dialogues is a “critical enabler” for an effective HIV response. Formal policy dialogues must be complemented by informal exchanges that take place over the course of a decade or more. The long-term collaboration between government, civil society, international organizations, academic researchers and the transwomen community was essential for the creation of a comprehensive HIV plan that is based on sound public health practices and human rights. We hope these elements will also help to promote a synergistic collaboration between the health system and community organizations and ultimately lead to the first comprehensive health policy (beyond HIV) for transwomen and other key populations in Peru.
